# The Use of Posterior Auricular Fascia Graft (PAFG) for Slight Dorsal Augmentation and Irregular Dorsum Coverage in Primary and Revision Rhinoplasty: A Prospective Study

**DOI:** 10.1007/s00266-023-03571-0

**Published:** 2023-08-25

**Authors:** Simone La Padula, Rosita Pensato, Chiara Pizza, Umberto Rega, Francesco D’Andrea, Giovanni Roccaro, Lucas Ungerer, Fabrizia Telesco, Luigi Canta, Benedetto Longo, Rasmieh Al-Amer, Jean-Paul Meningaud, Barbara Hersant, Edoardo Coiante

**Affiliations:** 1 Department of Plastic, Reconstructive and Maxillo facial Surgery, Henri Mondor Hospital, University Paris XII, 51 Avenue du Maréchal de Lattre de Tassigny, 94000 Créteil, France; 2 Department of Plastic and Reconstructive Surgery, Università degli studi di Napoli Federico II, Via Pansini 5, 80131 Napoli, Italy; 3 Department of Plastic and Reconstructive Surgery, Università di Roma Tor Vergata, Viale Oxford, 81, 00133 Roma, Italy; 4 Harvard University, Harvard Medical School, 25 Shattuck Street, Boston, MA 02115 USA; 5 Paris, France

**Keywords:** Rhinoplasty, Patient satisfaction, Rhinoplasty outcomes evaluation scale, Posterior auricular fascia, Nasal dorsal augmentation, Irregular dorsum coverage

## Abstract

**Introduction:**

Augmentation and coverage of irregularities of the nasal dorsum remain a challenge in rhinoplasty. Different techniques have been described in the current literature for this purpose. The aim of this study is to assess and illustrate the author experience and outcomes using the posterior auricular fascia graft (PAFG) for dorsal camouflage and augmentation in primary and revision rhinoplasty.

**Material and Methods:**

A prospective bicentric study was conducted, including patients with slight dorsal deficiencies and/or with dorsal irregularities following hump resection, trauma or previous rhinoplasty receiving PAFG to improve the rhinoplasty outcome. To objectively assess the graft resorption rate, MRI was performed 2 weeks and 18 months after surgery. To investigate patient satisfaction, the preoperative and 1-year postoperative scores obtained using the rhinoplasty outcomes evaluation (ROE) scale were compared. The scores following a normal distribution obtained for each patient were compared using a paired *t*-test.

**Results:**

Forty-five patients were enroled in this study. Average follow-up duration was 35.4 months. Patients’ age ranged from 17 to 57 years. No cases of infection or major graft resorption were observed. No postoperative scars were visible at the donor site. All patients were satisfied after surgery, and a statistically significant difference between pre- and postoperative scores (*p*<0.0001) was observed.

**Conclusion:**

This study showed that PAFG is a reliable technique for dorsal camouflage and slight augmentation in primary and revision rhinoplasty. The procedure is safe, easy and quick and only requires a small learning curve.

**Level of Evidence II:**

This journal requires that authors assign a level of evidence to each article. For a full description of these Evidence-Based Medicine ratings, please refer to the Table of Contents or the online Instructions to Authors www.springer.com/00266.

**Supplementary Information:**

The online version contains supplementary material available at 10.1007/s00266-023-03571-0.

## Introduction

Dorsal augmentation and smoothening of surface asperities are some of the challenges of rhinoplasty. Multiple surgical procedures may result into dorsal irregularities (hump resection, facial trauma or primary rhinoplasties). Dorsal asperities are described in 7–10% of primary rhinoplasties. Thin nasal dorsal skin would reveal and accentuate the uneven surface of the nasal dorsum resulting in low patient satisfaction [1, 2]. Trending techniques in rhinoplasty surgery are based on tailored grafts aimed at restoring nasal contour. Multiple grafts and implants have been described along with their relevant advantages and disadvantages. The best graft for minimal dorsal augmentation and irregular dorsum flattening should be easily available, bio-compatible and inexpensive. Moreover, the ideal graft should cause minimal donor site morbidity and carry a low risk of extrusion, shape loss, misplacement or infection. Depending on the degree of dorsum irregularities or dorsum deficiency, autografts, homografts and implants have been used for nasal reshaping and increment, including cartilage, dermis, temporal fascia, the SMAS, polyglactin 910 mesh, gelatin-based film, polytetrafluoroethylene, Surgicel-wrapped diced cartilage graft, fascia lata grafts and acellular dermis [3]. Diced cartilage grafts in autologous fascia are nowadays frequently used [4] and, in some cases, have replaced costal autologous grafts. Dermal fillers can be used to correct dorsal minimal irregularities and to obtain a slight dorsal augmentation. However, dermal fillers are resorbable (within 6–12 months), and there have been reports of potential risks, such as embolism and skin necrosis, associated with this procedure [5]. The aim of this study is to assess and illustrate the authors experience and outcomes using the posterior auricular fascia graft (PAFG) for nasal dorsum camouflage and slight augmentation in primary and revision rhinoplasty.

## Materials and Methods

A prospective bicentric study was conducted on patients undergoing elective rhinoplasty between November 2016 and November 2018. A division into primary and secondary cases was made, and the indications for the use of PAFG are given in Table 1. Patients presenting with thin dorsal skin, patients with residual dorsal irregularities after hump resection, saddle nose patients and patients who would benefit from a slight increase in the dorsal volume and projection were included in this study. Patients presenting with thick nasal skin or patients requesting dorsal projection over-augmentation and patients suffering from mental illnesses (such as bipolar disorder, personality disorder and anxiety disorder) were excluded from the study. The study was conducted in accordance with the globally accepted standards of Good Clinical Practice (ICH-E6) (European Directive 2001/20/EC), and the revised version of the Declaration of Helsinki set out in the European Directive. All the patients had to sign a written informed consent to enrol in the study between May 2016 and July 2018. Ethical approval was given by the French institutional committee with the relevant judgement’s reference number that is 2016-A214894-44. Patient’s selection was based on simple random sampling (95% of confidence interval and 5% of marginal error). The list of patients looking for rhinoplasty at both centres between November 2016 and November 2018 was sent to our statistician in charge of the randomization process. A total of 45 patients were included based on the sampling.

**Table 1:** Patients and study data.

	Primary procedures	Secondary procedures	P-value
Age (years)			
(Mean±Standard Deviation)	26.9 ± 20.1	27.8 ± 12.4	0.5
*Gender*			
Male	11	10	0.5
Female	12	12	0.5
*Follow-up (months)*			
(Mean±Standard Deviation)	34.9 ± 6.9	35.7 ± 7.8	0.5
Indications for the use of PAFG			
*Saddle nose*	3	0	n/a
Patients with thin dorsal skin (PAFG used to cover slight irregularities after hump resection)	15	19	0.5
Cases requiring a slight increase in volume and dorsal projection	5	3	0.5
Type of dorsum grafts (patients)	1	2	0.2
Dorsal onlay graft	6	21	0.00001
Septal spreader grafts	10	13	0.5
Septal extension grafts	1	1	0.5
*Dorsal augmentation with autogenous rib cartilage*			
Alar rim grafts	1	2	0.2
*Septal deviation type*			
C-shape	14	16	0.5
S-shape	7	3	0.1
No septal deviation	2	3	0.5
*Type of access*			
Open	17	22	0.008
Closed	6	0	n/a
Total ROE score	Preoperative 44.9	Preoperative 43.9	0.5
	Postoperative 90.2	Postoperative 90.6	0.5
	*P*-value 0.00001	*P*-value 0.00001	

n/a: not applicable.

### Surgical Procedure

To reduce bleeding, the donor site was infiltrated with a 0.25% bupivacaine with 1:200,000 epinephrine solution. A 4-cm line was traced above the auriculo-cephalic sulcus. The authors used a posterior auricular face-lift incision [6–8] in order to conceal the scars and to provide easy access to cartilage graft harvesting; in case, it was needed. Dissection was then carried out through the same incision of the retroauricular area, the skin was dissected off the underlying soft tissues 5–6 cm further. Blunt dissection in this plane (separating skin from fibroadipose tissue) was carried out (skin hooks retracted the flap). All the tissues under the skin were preserved accurately.

**Fig. 1:**
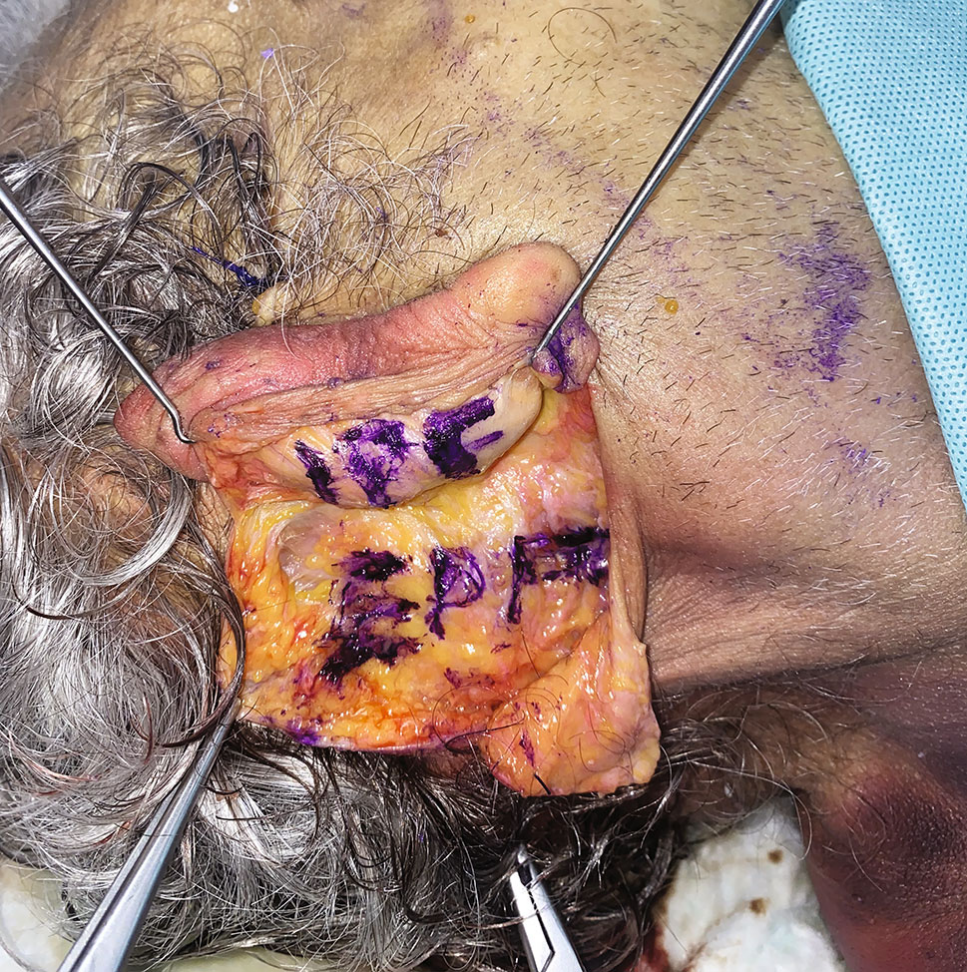
Cadaver dissection showing intrinsic postauricular fascia (IPF) and the thicker extrinsic postauricular fascia (EPF) continuous with the mastoid area. Auricular fascial incisura demarks the limits between IPF and EPF and corresponds with the surface anatomy of the auriculo-cephalic sulcus

Two layers of postauricular fascia should be distinguished: the intrisic postauricular fascia (IPF) and the extrisic postauricular fascia (EPF) [9]. IPF includes the fibroadipose and vascular tissue, nerves and perichondrium envelope to the ear. It continues over the auriculo-cephalic sulcus into the EPF. EPF is a thick, multi-layered fascia, showing a prevalent fibrous component and connected to the posterior auricularis muscular fibres. EPF (Fig. 1) was accurately dissected through blunt and sharp dissection to the mastoid area. Accurate haemostasis was carried out, all the incisions were stitched using Vicryl RapideTM 4-0 sutures. Drains were not applied, and light compression was applied to the wound. The nose dorsum was accessed through an open or closed rhinoplasty approach. Dorsal preservation techniques were not included in this study.

In most cases, the following procedures were performed: For open rhinoplasty, the initial step involved making a trans-columellar incision, followed by marginal incisions. For septoplasty, an extramucosal dissection of the septum was employed, followed by osteo-cartilaginous hump removal and lateral osteotomies using the low-to-low technique. Nasal tip sculpting was then performed, and the final step involved the placement of PAFG. In cases where a closed rhinoplasty was performed, an intersepto-columellar incision was made to access the nasal septum. The same manoeuvers as those used in open rhinoplasties were employed for septoplasty and osteotomies. For more complex cases requiring nasal tip reshaping, the delivery technique was used to ensure full visibility of the lower lateral cartilages. In simpler cases, a trans-cartilaginous incision was made to perform cephalic trim of the lower lateral cartilages. The final step of the procedure involved the placement of PAFG. The dissection plane was obtained by blunt dissection of the dorsum. In all cases, dorsum undermining was minimal in the sub-SMAS plane to create a custom-made recipient site for the graft and to secure stable insetting and reduce misplacement.

Based on the length of the nasal dorsum, the PAFG was cut with blunt scissors to fit the entire dorsum. The cephalic segment of the fascia was anchored through two Vicryl RapideTM 4-0 stitches. These sutures pierced the proximal segment of the fascia and the nasal skin at a nasal root level and were knotted together in order to stabilize the graft. Definitive tailoring and positioning of the graft were performed by external digital moulding (Video 1). The distal segment of the graft was fixed at supra-tip level through Vicryl rapid 4/0 sutures.

These sutures pierced the distal segment of the fascia and the nasal skin at supra-tip level.

**Fig. 2:**
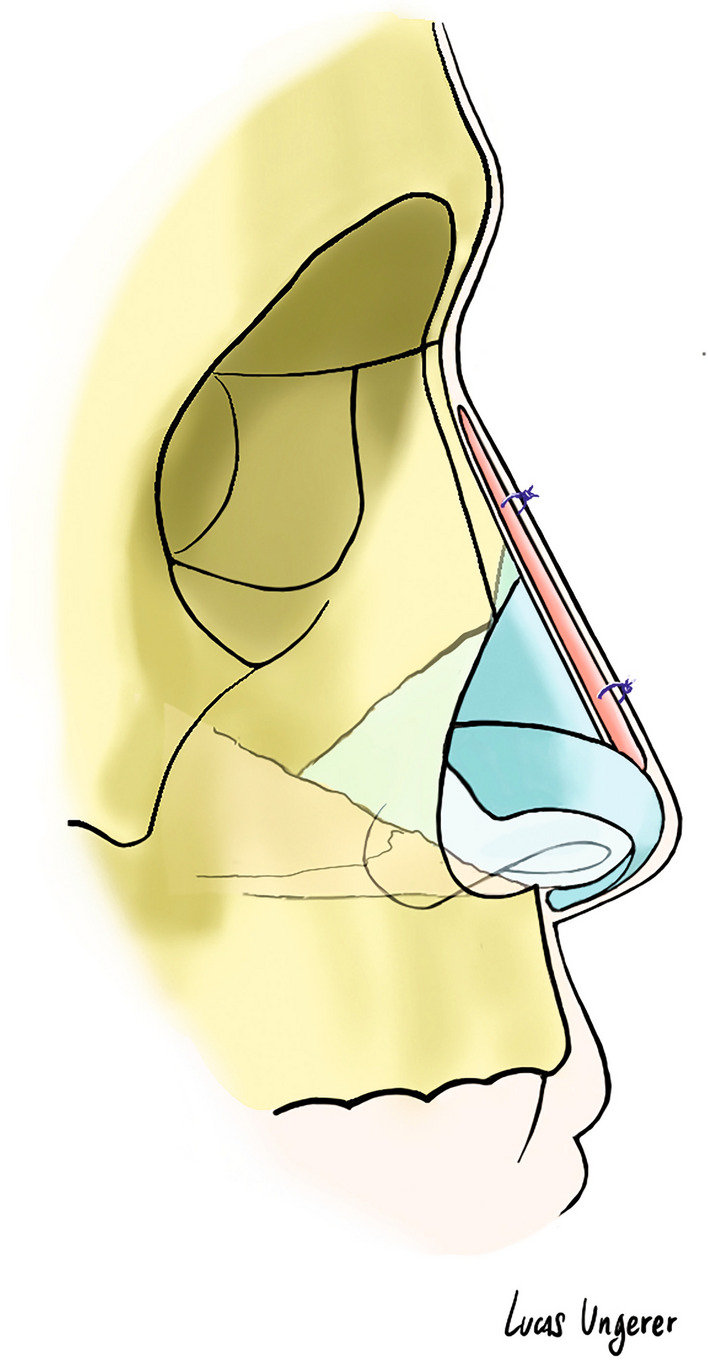
Schematic representation of the PAFG positioning

A fragment of greased gauze was placed under the proximal and distal knot to reduce pressure sores over the dorsum (Fig. 2). These sutures (proximal and distal) were removed 3 weeks after surgery. When only volume augmentation was required, a double-layer folded fascia graft (double-layer PAFG) was used; the insetting procedure was the same, with Vicryl sutures passing the two layers of fascia cranially and caudally. In cases where an autologous cartilage graft was used for dorsal augmentation (such as rib cartilage), the fascia was sutured to the dorsal surface of the cartilage graft. Steri-Strips (3M, St. Paul, Minn.) and tailored splints were then placed over the nasal pyramid to help maintain the graft in situ, so that the nose would heal into the desired shape.

A 5-day postoperative antibiotic prophylaxis course was prescribed. Splints were removed after 10 days. All surgeries were performed by the first author (SLP) at both teaching hospitals. Objective measurements of the graft resorption rate were performed through postoperative magnetic resonance imaging (MRI) scans (2 weeks and 18 months after surgery). PAFG thickness was considered by radiologists as a reliable measure of graft resorption.

Based on the recommendations of our radiologists, it is advisable to evaluate graft resorption at a minimum of 18 months after surgery. MRI scans can provide more accurate information in this regard. If resorption has not occurred after 18 months, it is unlikely to happen at a later stage.

### Patients Satisfaction Assessment

Patient satisfaction and quality of life improvement were assessed through the rhinoplasty outcomes evaluation scale. The ROE questionnaire [10–12] consists of a six sectioned questionnaire assessing both functional and aesthetic outcomes. Patient provides answers ranging from 0 to 4, providing a total score of 24, which is then converted to a percentage. This questionnaire consists of the following questions describing three quality of life domains: physical, mental/emotional and social:

1. How well do you like the appearance of your nose?

2. How well are you able to breathe through your nose?

3. How much do you feel your friends and loved ones like your nose?

4. Do you think your current nasal appearance limits your social or professional activities?

5. How confident are you that your nasal appearance is the best that it can be?

6. Would you like to surgically alter the appearance or function of your nose?

Total scores were divided by 24 and then multiplied by 100 to give a satisfaction score on a scale of 0–100. For questions 1, 2, 3 and 5, a score of 0 means “not at all” and 4 means “completely”. For question 4, 0 means “always” and 4 means “never”. For question 6, a score of 0 means “definitely” and a score of 4 means “no”. Questionnaires were taken preoperatively before surgery and 1 year post-procedurally. Data collected from pre- and postoperative questionnaires were analysed.

### Statistical Analysis

The normality of the data of the first phase was assessed by the Kolmogorov–Smirnov test. Quantitative data were reported as mean ± standard deviation (SD). Considering the data normality, a paired t-test was used to compare variables preoperatively and postoperatively. A p value of < 0.05 was statistically significant. All the authors were responsible for the integrity of the data. Analyses were performed using PRISM, version 7 (GraphPad, USA). Two medical statisticians analysed the outcomes.

## Results

**Fig. 3:**
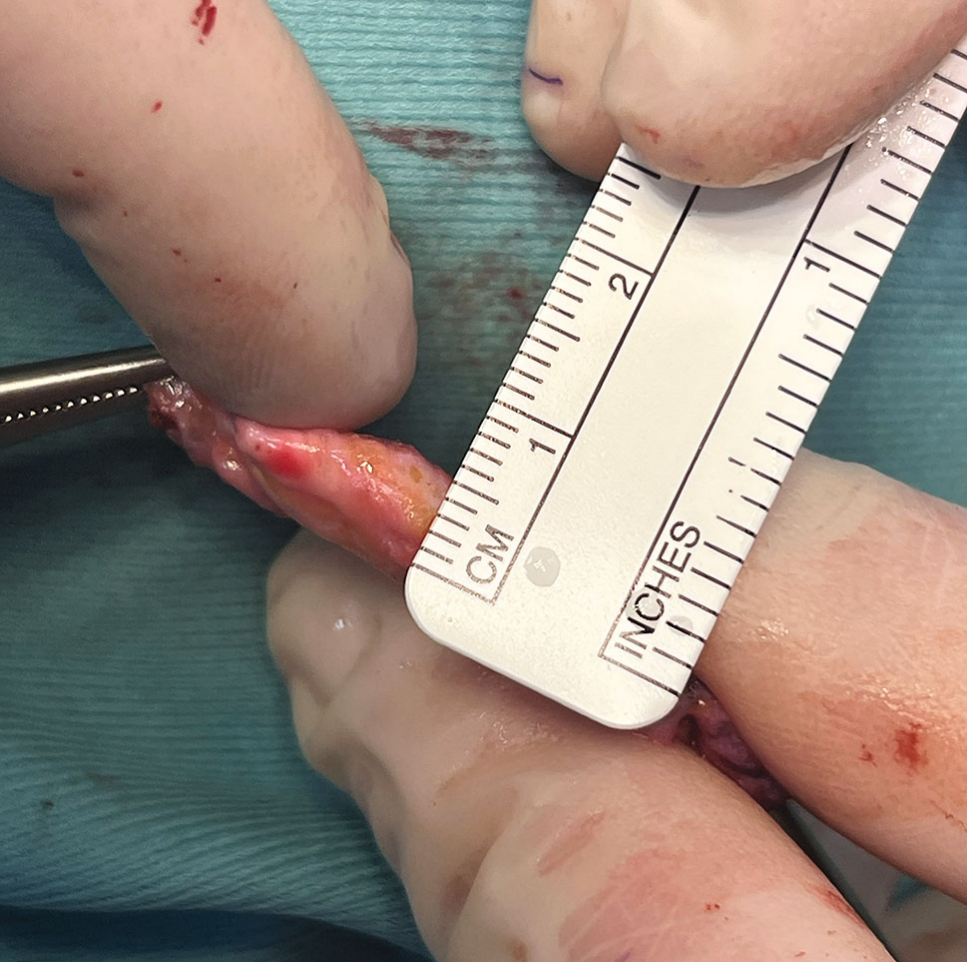
This image depicts the intraoperative appearance of the posterior auricular fascia graft, which measured 5 mm in thickness in this particular case. In our study, the mean thickness of the postauricular fascia was found to be 3.5 ±2.7 mm (range of 3.5-6.2 mm). These findings suggest that the thickness of the PAFG may vary among individuals and should be carefully considered during surgical planning.

**Fig. 4:**
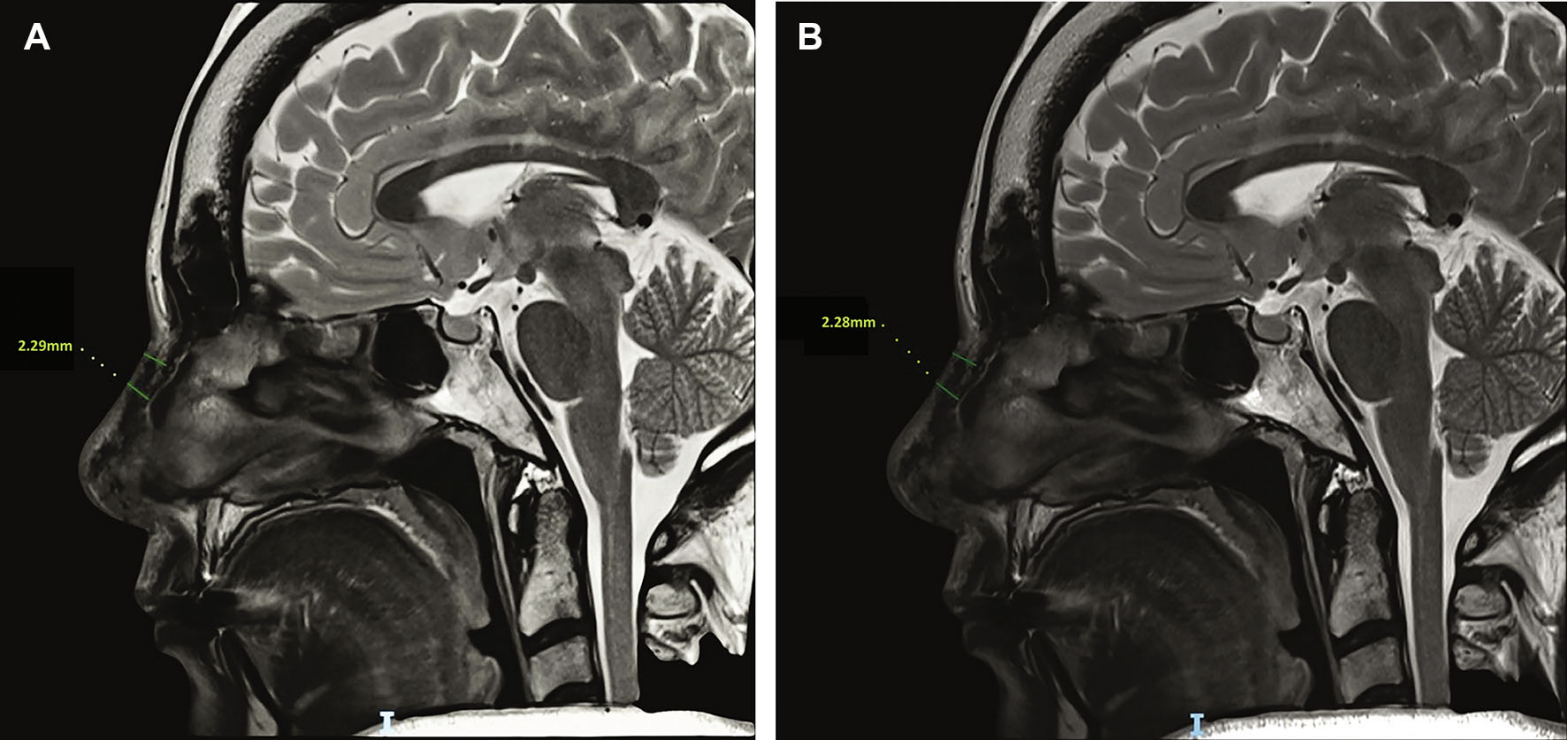
Example of a 2-week postoperative MRI **a**. The fascia construct maintains its volume and shape throughout the postoperative period and as confirmed by the 18 months postoperative MRI **b**, no major graft resorption occurred in our series

**Fig. 5:**
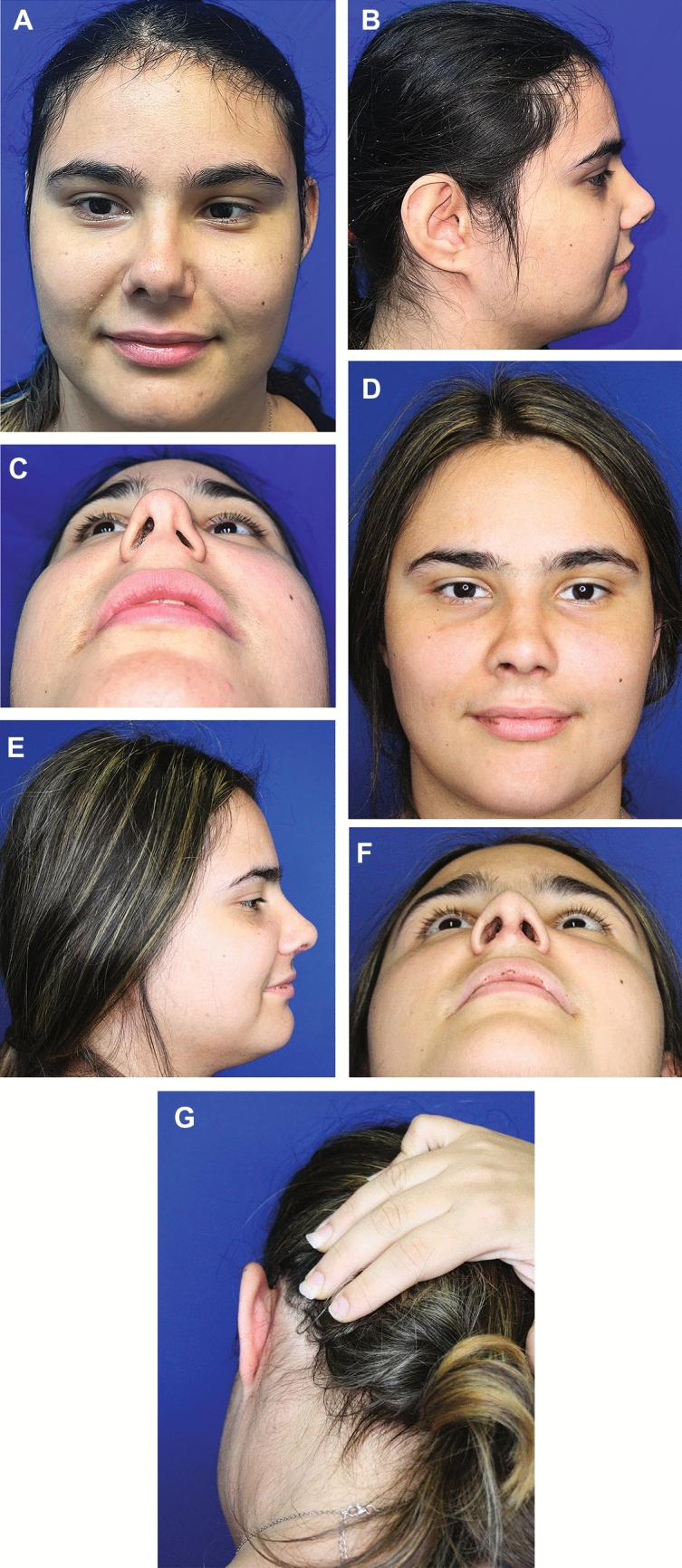
Preoperative appearance of a 20-year-old girl **a**-**c**. The patient immediately after birth had a nasal catheter inserted, which caused adhesion of the left soft triangle with collapse of the external nasal valve. She approached us because she wanted a slight lowering of the nasal tip, correction of a mild dorsal irregularity and correction of the collapse of the left external nasal valve. The patient underwent open rhinoplasty. A septal extension graft taken from the nasal septum was used to slightly rotate the nasal tip downward. Postauricular fascia was employed to correct the mild dorsal irregularity, and the same retroauricular incision used for harvesting the fascia was used to harvest a conchal cartilage graft to correct the collapse of the left external nasal valve. Residual nasal mucosa excess at the end of the procedure was used to correct the iatrogenic adhesion of the left soft triangle. The patient was highly satisfied with the achieved result **d**-**f**. The postauricular scar was well hidden and did not cause any problems for the patient **g**

**Fig. 6:**
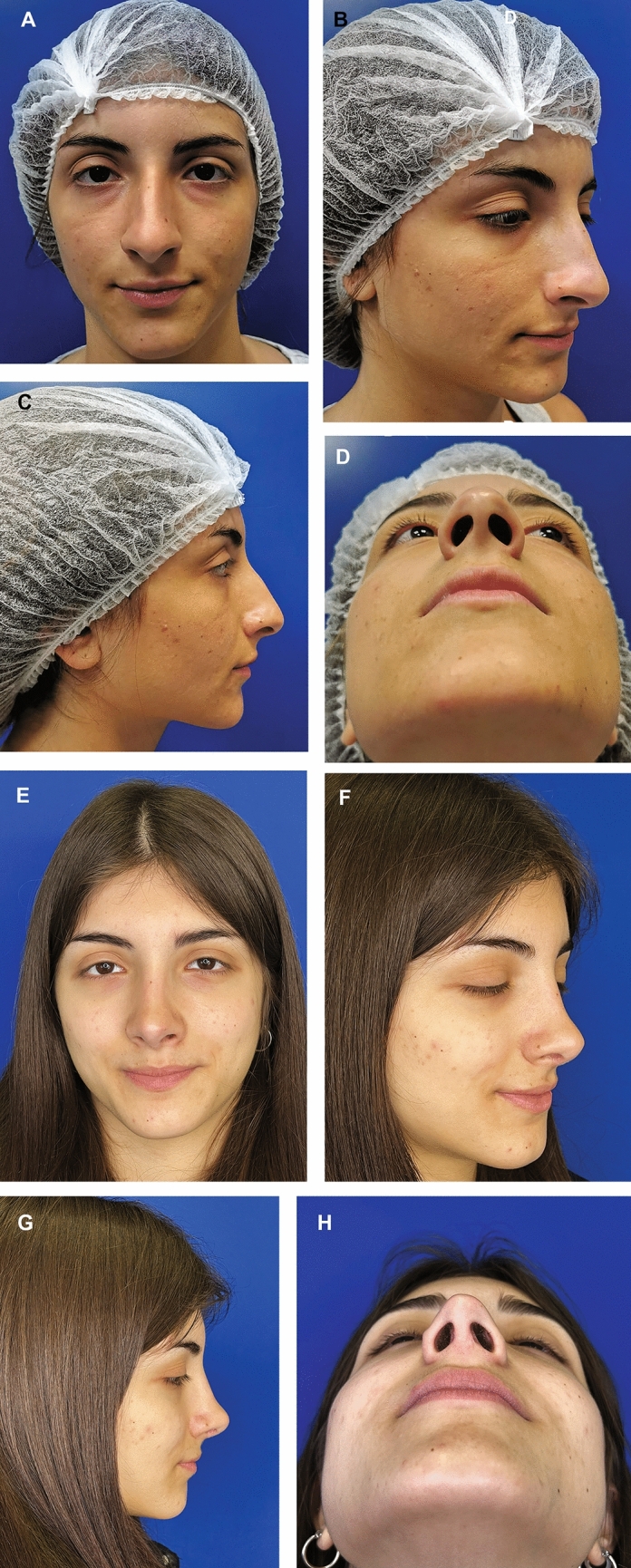
Preoperative photographs of a 28-year-old woman who underwent primary aesthetic and functional rhinoplasty using an open approach **a**-**d**. The procedure included tip reshaping with the use of a septal extension graft and intradomal sutures. The postauricular fascia graft (PAFG) was used to address the remaining irregularities on the nasal dorsum after the removal of the osteo-cartilaginous hump. During the 1-year follow-up, the patient expressed high satisfaction with the outcome and reported no irregularities on the nasal dorsum **e**-**h**

Forty-five (21 males and 24 females) patients were included in this study (Table 1). The average follow-up time was 35.4 ± 7.6 months (ranging from 23 to 43 months). Age ranged from 17 to 57 years (average, 27.4 ± 19.3 years). The mean surface of the harvested fascia graft was 5.0 ± 0.67×3 ± 0.4 cm with a mean thickness of 3.5 ±2.7 mm (range 3.5–6.2 mm) (Fig. 3). The mean PAFG dissection time was 10 ± 3.67 minutes. A single-layer PAFG was used in thirty-nine cases, while six patients received a double-layer PAFG. No cases of infection or major graft resorption were clinically observed. Haematoma, occurred at the donor site in one patient, was surgically evacuated. No lateral displacement of the fascia was observed until the last follow-up, and no patient required revision surgery. Only one patient who received a double-layer PAFG benefited from external manual repositioning of the graft after splint removal to improve its insetting 10 days postoperatively in the outpatients setting. This gentle manoeuver was carried out without anaesthesia and was painless. Two weeks after surgery, healing was completed, and we believe that at this point, it is no longer possible to shape the graft any further. About 100% of the grafts maintained their volume and shape postoperatively. PAFG stability over time was proved by 18 months postoperative MRI (Fig. 4): The mean PAFG thickness 2 weeks postoperatively was 3.29 ± 2.5 mm and 3.28 ± 2.3 mm 18 months postoperatively for patients who had received a monolayer PAFG (*p *= 0.5). The mean PAFG thickness 2 weeks after surgery was 6.28 ±1.2 mm and 6.27 ±1.2 mm 18 months after surgery for patients who had received double-layer PAFG (*p *= 0.3). Patient satisfaction was high (Figs. 5 and 6; Table 2). The overall 1-year post-procedurals ROE mean scores (90.4 ± 11.13) were significantly higher than the pre-procedural mean scores (46.6 ± 12.65), demonstrating significant post-procedural improvements in quality of life (*p* < 0.0001). Satisfaction with the procedure was high for both primary and secondary rhinoplasty cases (*p*<0.0001) (Table 1).

**Table 2:** Overall mean pre- and postoperative scores from questions 1 to 6 on the rhinoplasty outcomes evaluation (ROE).

Question	Preoperative	Postoperative	*P*-value
1	1.2	3.7	0.00001
2	2.7	3.6	0.00001
3	2.9	3.4	0.00001
4	2.1	3.9	0.00001
5	1.4	3.5	0.00001
6	0.9	3.6	0.00001
Total ROE score (0–100)	46.6	90.4	0.00001

## Discussion

Until today, several techniques for dorsal irregularities correction and augmentation have been described [13–25]. This is the first study to date using PAFG alone for dorsal augmentation in rhinoplasty. Until now, PAFG has exclusively been employed as a composite graft in conjunction with diced cartilage, and there has been a lack of objective studies examining its reabsorption over time. To address nasal dorsal irregularities and achieve dorsal augmentation, techniques involving the use of diced cartilage wrapped in autologous or alloplastic materials have been described thus far. Turkish delight [16] was based on diced autologous cartilage mixed with blood with Surgicel wrapping (Johnson and Johnson, Somerville, N.J.). This technique was acquired by Daniel, Calvert and Aiach [17–23] who preferred autologous fascia wrapping for the cartilage diced grafts. Blood was cleared from cartilage grafts. Compared to Surgicel which was proven to resorb within 24 hours, autologous fascia was proven to be a stable graft with high tensile strength [17]. Moreover, fascia is known to have scaffolding properties to cells and enhances graft incorporation in the recipient site [4, 23]. Very shortly after Daniel, Calvert and Aiach reports, hybrid grafting started to be very commonly used. Nevertheless, drawbacks of this technique are the relatively long operative time (fascia harvest, cartilage harvest and construct preparation) and the risk of possible residual alopecia following the harvesting of the temporal fascia [24]. Our results have demonstrated that PAFG is a safe, reliable and viable alternative to crushed cartilage in achieving mild dorsal augmentation and correcting minor irregularities of the nasal dorsum. Furthermore, there is no risk of alopecia, and the harvesting technique is simpler compared to that of the temporal fascia, as the skin in the postauricular region is more elastic. The PAFG is an invaluable option for dorsum contouring. It is a versatile graft that can easily dissimulate contour irregularities. It also allowed the creation of a “soft” light-shadow limit between the dorsal and lateral nasal subunits (we observed an improvement in the dorsal aesthetic lines, making the boundary between the dorsal and lateral subunits more discernible). In our varied patient cohort, very satisfactory long-term results were observed. Our results allow us to suggest the use of PAFG for patients with thin dorsal skin, residual dorsal irregularities post-hump resection, saddle nose patients, patients requiring a slight increase in dorsal volume and projection and those who had previously undergone rhinoplasty but still had visible dorsal irregularities. Thin dorsal skin can pose a challenge for rhinoplasty surgeons, as the underlying cartilage framework may be visible or palpable after surgery. PAFG use in our patients provided additional coverage to the nasal dorsum, concealing the underlying cartilage and creating a smooth, natural-looking nasal contour. Patients with post-traumatic saddle nose, who had lost dorsal support and projection, experienced significant benefits from the use of PAFG. The graft successfully restored the height and projection of the nasal dorsum, resulting in high patient satisfaction with the aesthetic outcome of the surgery. In our study, we also noted a high level of satisfaction among patients who only sought a slight increase in dorsal volume and projection for aesthetic reasons. In particular, when only volume augmentation was needed, a folded and double-layered fascia graft was used (double-layered PAFG). This variant would shorten the operative time required when diced cartilage wrapped in an autologous fascia must be prepared. Mean PAFG thickness was of 3.5 ± 2.7 mm so a double-layered PAFG could be sufficient to increase the volume of the nasal dorsum by approximately 6 mm. The fascia graft volume is maintained stable over time according to the 18 months post-procedural MRI. This study demonstrates that the PAFG is an invaluable technique for dorsum camouflage and augmentation in primary and revision rhinoplasty. A small learning curve is required to integrate this technique into a plastic surgery specialist arsenal. The ease and flexibility of this procedure should accelerate its integration into the standard rhinoplasty techniques. Although we obtained encouraging results, we recognize that our study may have some limitations: small sample size, experience of a single surgeon, follow-up period (even if our study has shown a stability over time of the graft, as confirmed by the use of MRI, there could be a late resorption of the same). Furthermore, an important limitation of our study methodology concerns the evaluation of aesthetic outcomes, as in cases where PAFG was used to correct irregularities of the nasal dorsum remaining after hump resection, it is difficult to estimate the real aesthetic benefit that patients have actually obtained from the use of postauricular fascia graft. Other limitations include the indications for the use of PAFG in rhinoplasty. The availability of tissue is restricted due to the limited amount of posterior auricular fascia that can be harvested, which can constrain cases requiring larger amounts of grafting material. The thickness of the posterior auricular fascia may also be compromised, varying with the patient's age, health and individual differences, which may impact the procedure's outcome. In our experience, we have observed that younger patients tend to have thicker fascia compared to older patients. Moreover, issues with grafting take may arise leading to infections, underscoring the importance of patient selection. Patients with diabetes and smokers, for instance, may experience graft survival issues and poorer scarring at the fascia harvesting site. Other possible complications include persistent discomfort or pain, haematoma, infection and nerve damage at the donor site. As such, the decision to use PAFG in rhinoplasty should be made on a case-by-case basis, considering the patient's unique circumstances and needs, following a thorough evaluation. Therefore, we encourage the scientific community to conduct further studies on this topic with a larger number of patients and a longer follow-up.

### Supplementary Information

Below is the link to the electronic supplementary material.

PAFG harvesting and insetting. PAFG harvesting is easy and safe and takes about 10 minutes (MOV 75,023 kb).
